# Bioaccumulation of Pyraoxystrobin and Its Predictive Evaluation in Zebrafish

**DOI:** 10.3390/toxics10010005

**Published:** 2021-12-24

**Authors:** Wenzhu Wu, Jing Xu, Yezhi Dou, Jia Yu, Deyang Kong, Lixiang Zhou

**Affiliations:** 1 College of Resources and Environmental Sciences, Nanjing Agricultural University, Nanjing 210095, China; lxzhou@njau.edu.cn; 2 Key Laboratory of Pesticide Environmental Assessment and Pollution Control, Nanjing Institute of Environmental Sciences, Ministry of Ecology and Environment of the People’s Republic of China, Nanjing 210042, China; xujing@nies.org (J.X.); douyezhi@126.com (Y.D.); yujia@nies.org (J.Y.); kdy@nies.org (D.K.)

**Keywords:** pyraoxystrobin, zebrafish, bioconcentration, model prediction

## Abstract

This paper aims to understand the bioaccumulation of pyraoxystrobin in fish. Using a flow-through bioconcentration method, the bioconcentration factor (*BCF*) and clearance rate of pyraoxystrobin in zebrafish were measured. The measured *BCF* values were then compared to those estimated from three commonly used predication models. At the exposure concentrations of 0.1 μg/L and 1.0 μg/L, the maximum *BCF* values for pyraoxystrobin in fish were 820.8 and 265.9, and the absorption rate constants (*K*_1_) were 391.0 d^−1^ and 153.2 d^−1^, respectively. The maximum enrichment occurred at 12 d of exposure. At the two test concentrations, the clearance rate constant (*K*_2_) in zebrafish was 0.5795 and 0.4721, and the half-life (*t*_1/2_) was 3.84 d and 3.33 d, respectively. The measured BCF values were close to those estimated from bioconcentration predication models.

## 1. Introduction

**Figure 1:**
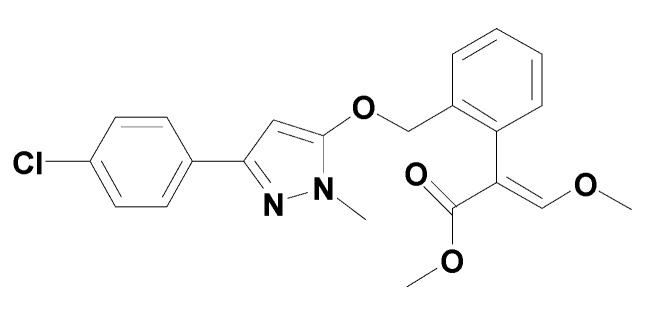
The structural formula of pyraoxystrobin.

Pyraoxystrobin is a broad-spectrum fungicide developed by the Shenyang Research Institute of Chemical Industry [1,2]. Its chemical name is (E)-2-(2-((3-(4)-chlorophenyl)-1-methyl-1H-pyrazole-5-oxy)methyl)phenyl)-3-methoxymethyl acrylate, with the molecular formula of C_12_H_21_ClN_2_O_4_, relative molecular weight of 412.9, and structural formula as follows (Figure 1):

The original fungicide compound is a white crystalline solid, easily soluble in dimethylformamide, acetone, ethyl acetate, methanol; slightly soluble in petroleum ether; and insoluble in water (0.13 mg/L). The hydrolysis rate at pH4 and pH7 is less than 10%, and it is chemically stable under weak acid (pH4) and neutral (pH7) conditions. As a member of the strobilurin fungicide, pyraoxystrobin has certain activities against fungal spores. Its mechanism of action is to prevent cell ATP synthesis through electron transfer between cytokines b and c1, thereby inhibiting its mitochondrial respiration. Pyraoxystrobin can inhibit the growth of hyphae and the germination of spores. It has a good inhibitory effect on subphylum deuteromycotina, flagellum subdivision, and ascomycotina. It is also effective on cucumber powdery mildew and downy mildew [3,4].

Bioaccumulation is often used to describe the process by which aquatic organisms accumulate chemicals through non-dietary means. It is an important indicator during the aquatic ecotoxicological evaluation of new pesticides, as well as hazard evaluation of residual pesticides to environmental organisms and the ecosystem. In general, the stronger the bioaccumulation capacity of pesticides is, the greater the potential chronic harm to organisms will be. Pyraoxystrobin is hydrolytically stabile and has a hydrolysis half-life of 577.5 days [5,6,7], showing high toxicity in different life stages of zebrafish [8]. Studies have shown that daphnia are sensitive to pyraoxystrobin, which can lead to heart rate decline, hatching inhibition, growth degradation, and morphological abnormalities. It can also change the mRNA levels of genes in zebrafish embryos [7,8]. Since pyraoxystrobin has a high lipophilicity (log Kow is 4.37) [5], it is important to study its potential bioaccumulation and provide direct scientific data to its ecological safety assessments.

## 2. Materials and Methods

### 2.1. Materials

Instrument and equipment: high-performance liquid chromatography-triple quadrupole tandem mass spectrometer (Agilent Technologies 1290 Infinity-AB SCIEX Triple Quad 4500, Santa Clara, CA, USA), Ultra-Turrax^®^ IKA^®^ T18 basic homogenizer (IKA, GER), Excella E24R full temperature oscillator (New Brunsuick Scientific, Edison, NJ, USA); CR 22GIIcentrifuge (HITACHI, Tokyo, Japan); Rotavapor R-210 rotary evaporator (BUCHI, SW); MG-2200 nitrogen-blowing apparatus (EYELA, Tokyo, Japan); flow-through water fish toxicity test device (Beijing Esen Technology Development Co., Ltd. Beijing, China).

Reagents: acetonitrile and formic acid are both chromatographically pure (TEDIA, Columbus, OH, USA), sodium chloride (baked at 140 ℃ for 4 h), anhydrous sodium sulfate (baked at 130 ℃ for 4 h), dichloromethane (analytical reagent, Nanjing Chemical Reagent Co., Ltd., Nanjing, China), and the experimental water was Milli-Q ultrapure water (Millipore Corporation, New England, MA, USA).

Organism being tested: zebrafish (*Brachydanio rerio*). At the beginning of the experiment, the fish had a body length of 2 to 3 cm (with tail), with a weight of 0.27 to 0.30 g, and an average fat content of 3.48%. Before the test, they were domesticated indoors for 1 week. During this period, they were fed normally, and oxygenated day and night. The water temperature was (23 ± 1) °C, and the pH value was 7–8. The zebrafish grew normally in the pre-raising period, free of disease, without visible deformities and deaths.

The purity of the pyraoxystrobin standard product is 98.0%.

### 2.2. Determination of Test Concentration

According to the report by Yang [9] report, the LC_50_ of pyraoxystrobin is 5.0 µg/L^−1^, and the previous fish acute toxicity test (96 h) results in our own laboratory showed that the LC_50_ of pyraoxystrobin was 10 µg/L^−1^. Both results had high toxicity to *Brachydanio rerio*. According to the acute toxicity test data (LC_50_ = 10.0 μg/L, 96 h), two concentrations, 0.1 μg/L and 1.0 μg/L, were selected for the study. Three replicates were set up for each treatment, and blank control and solvent blank control treatments were used.

### 2.3. Flow-Through Bioconcentration Test

The flow test device consists of a peristaltic pump, two balances with different precisions and ranges, two-stage and three-stage stock solution containers, a test solution storage, a test tank, and an automatic control system. The stock solution of the test substance was diluted to obtain two test solutions of different desired concentrations. Within a certain time-interval, we began solution preparation in the preparation tank to ensure that there was enough test solution in the storage tank to be continuously pumped into the test tank. At the same time, the flow rate of the test solution in the storage tank was consistent with the update rate of the test substance solution in the test tank. During the preparation of the test solution dilution, magnetic stirring was continued to ensure that the test solution was evenly mixed, and all the dilution processes were automatically completed by the flow-through test device.

### 2.4. Preparation of the Stock Solution of the Test Substance

An accurate amount of 0.1042 g pyraoxystrobin was weighed into a 100 mL volumetric flask and diluted to the mark with acetone to obtain a 1000 mg/L pesticide standard working solution for standby use. The standard working solution was processed by using the multiple dilution method to obtain the stock solution of the test substance (first level) at a concentration of 40 mg/L.

### 2.5. Automatic Preparation of Test Solution

The reagent bottle (1000 mL) containing the stock solution (first stage) of the test substance was placed in the stock solution rack of the flow-through test device. Then, the stock solution was diluted proportionally with a peristaltic pump and a balance, and a first-stage dilution ratio of 1:100 was set to obtain a test substance solution with a concentration of 400 μg/L. The second-stage dilution ratio was 1:40, from which a test substance solution with a concentration of 10.00 μg/L was obtained. Then it was diluted step by step according to the dilution ratio of 1:10, and finally, the test substance solutions were prepared with concentrations of 1.0 μg/L and 0.1 μg/L, respectively. In the final test solution, the acetone content of the solvent used was 0.0025%.

In this project, the update frequency of the test substance solution was set to 8 h. In order to ensure stable operation of the system, the flow-through test device was pre-run for 24 h before the start of the test. During the pre-run period, samples of the test solution were collected at 8 h and 24 h, respectively to determine the test substance content in the test solution to check the stability of the test substance solution concentration in the test medium. During the test, the measured average flow rate of the test substance solution in the test cylinder was 12.5 (±1.20%) mL/min (the test solution update rate is about 8 h).

### 2.6. Exposure Conditions

All tested fish were zebrafish from the same source at the same age. The fish body length (with tail) is (2.0–3.0) cm, and the weight is (0.2 ± 0.1) g. In each test tank with 6 L test solution, we put 40 pieces of zebrafish, the average fish carrying a capacity of about 1.0 g/L. During the test period, the same food as that during the domestication period was used. The fish were fed with bait equivalent to 1% to 2% of their body weight every day. Within 30 to 60 minutes after daily feeding, we siphoned the remaining bait and fish excrement in the test tank. Throughout the entire test period, we kept the test cylinder as clean as possible to ensure that the concentration of organic matter was as low as possible. During the test, 16 h of light were ensured every day. The room temperature and the temperature of the water bath was kept at (23 ± 2) °C. The duration of the absorption phase was 28 d, and the results of fish body measurements indicated that equilibrium had been reached. The duration of the cleanup phase was 10 d. The test solution was tap water filtered by an activated carbon filter, aerated and dechlorinated without the test substance, and normal feeding was conducted during the test.

### 2.7. Sample Collection

Fish and water samples were collected regularly to determine the concentration of pyraoxystrobin in the fish body and test solution at 3 d, 7 d, 12 d, 18 d, 22 d, and 28 d. Meanwhile, water quality parameters, such as dissolved oxygen and the pH value of the test solution, were regularly measured.

### 2.8. Sample Extraction and Determination Method

Water sample extraction: Samples at multiple locations in the test system were taken and mixed. Of the water sample, 20 mL was poured into the separatory funnel, with 20 mL of dichloromethane added, and the sample was then shaken and the organic phase collected after phase separation. The aqueous solution was extracted once more with 20 mL of dichloromethane. We combined the organic phases, evaporated to near dryness, blow-dried with N_2_, diluted to volume with acetonitrile, filtered through a 0.22 μm filter membrane, and waited for LC-MS determination.

Fish sample: We took 4 weighed fish, added 10 g of anhydrous sodium sulfate, ground them into powder, and placed them in a triangular flask, added 10.0 mL of acetonitrile, placed on a shaker and shook for 30 minutes, and then ultrasonically extracted for 30 minutes. After the extract was diluted to the concentration of the injection standard sample sequence, it was filtered through a 0.22 μm filter membrane and then determined by LC-MS.

HPLC determination conditions: ZORBAX Eclipse Plus C_18_ 3.5 μm, 2.1 mm × 150 mm column was used with a mobile phase of acetonitrile −0.1% formic acid aqueous solution. The flow rate was 0.4 mL/min, and injection volume was 10 μL. The column temperature was 40 °C. The gradient elution program is shown in Table 1.

Mass spectrometry conditions: ion source mode: electrospray (ESI) ion source, negative ionization mode, curtain gas (CUR): 241.3 kPa, spray gas (GS1): 344.8 kPa, auxiliary heating gas (GS2): 379.2 kPa, source temperature (TEM): 450 °C, ionization voltage (IS): 4500 V, collision energy (CE): 25 V, de-clustering voltage (DP): 90 V, inlet voltage (EP): 10 V, export voltage (CXP): 7 V, precursor mass-to-charge ratio: 413.40, daughter ion mass-to-charge ratio: 205.200, retention time: about 1.30 min.

Method recovery rate: when the water addition level was 0.05~1.0 μg/L, the average recovery rate was 92.0~94.0%, and the *RSD* was 1.6~3.3%; when the addition level of the standard solution in the fish body was 0.05~1.0 mg/kg, the average recovery rate was 72.3~105.7%, and the relative standard deviation (*RSD*) was 3.3~7.4%. The minimum detection concentration of pyraoxystrobin was 0.02 μg/L, the minimum detection concentration of fish was 1.0 μg/kg, and the minimum detection concentration in water was 0.05 μg/L.

### 2.9. Data Analysis

We took the fish body concentration or *BCF* value at each time point as the ordinate and drew the curve with the sampling time as the abscissa. The curve reached a plateau, that is, where it was almost parallel to the abscissa (with a variation range of ± 20%), and the steady-state *BCF*_ss_ value was calculated as follows:

(1) $${BCF}_{\mathrm{ss}}=\frac{C_{f}(steady-state)}{C_{w}(steady-state)}$$

In the formula: *C_f_*: the average concentration of the test substance in the fish body tissue; *C_w_*: the average concentration of the test substance in the test solution.

Test substance clearance rate: Clearance rate = *C_f_/C*_0_. *C_f_* is the concentration of the test substance in the fish body at t d (μg/kg), while *C*_0_ is the concentration of the test substance in the fish body at 0 d.

Absorption rate constant *K*_1_

In the absorption rate constant: $K_{1}=\frac{C_{f}K_{2}}{C_{w}\times (1-e^{-K_{2}t})}$, *C_f_*: is the concentration of the test substance in the fish body at *t* d (μg/kg); *C_w_* is the concentration of the test substance in the water (μg/L); *t* is the time (d); and *K*_2_ is the removal rate constant.

Clearance rate constant *K*_2_:

In the clear rate constant: K2=ln(Cf1Cf2)t2−t1, *C_f_* is the concentration (μg/kg) of the test substance in the fish body on *t* d; *t* is the time (d).

Dynamic bioconcentration coefficient (*BCF*_k_): The dynamic bioconcentration coefficient *BCF*_k_ = *K*_1_/*K*_2_, *K*_1_ is the absorption rate constant; *K*_2_ is the clearance rate constant.

### 2.10. Statistical Analysis

SPSS 19.0 was used for data processing and correlation analysis.

## 3. Results

### 3.1. Concentrations of Tested Solution and Fish Body

During the test, the updated flow rate of the tested solution was 12.5 (±1.20%) mL/min and the flow rate change rate was less than 10%. In the absorption stage, for the test solution with a concentration of 0.1 μg/L, the measured concentration of the test substance was 0.091~0.10 μg/L, and the average concentration was 0.094 μg/L. For the test solution with a concentration of 1.0 μg/L, the measured concentration of the test substance was 0.912~0.989 μg/L, and the average concentration was 0.94 μg/L. The concentration of the test substance remained near constant (the concentration change was less than 20% of the initial measured concentration, see Table 2). Meanwhile, the measured concentrations in the fish body were 41.3~79.5 μg/kg and 191.6~260.8 μg/kg, respectively.

### 3.2. Fish Bioaccumulation of Pyraoxystrobin

**Figure 2:**
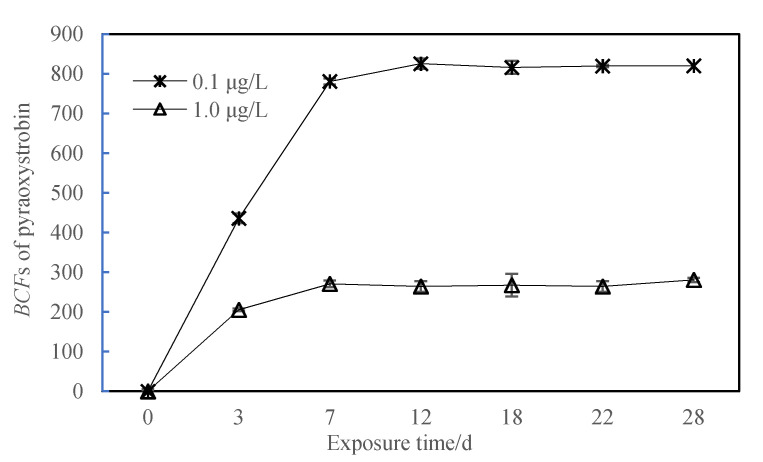
The *BCF* value of pyraoxystrobin in zebrafish.

Results of the exposure phase test (Figure 2) showed that under the exposure conditions of pyraoxystrobin, the *BCF* of zebrafish reached a steady state on approximately Day 12. Based on the concentration of the test substance in the test solution, the steady-state *BCF*_ss_ was calculated as 820.8 (714.8~925.3, 0.1 μg/L) and 265.9 (189.7~344.5, 1.0 μg/L), respectively.

**Figure 3:**
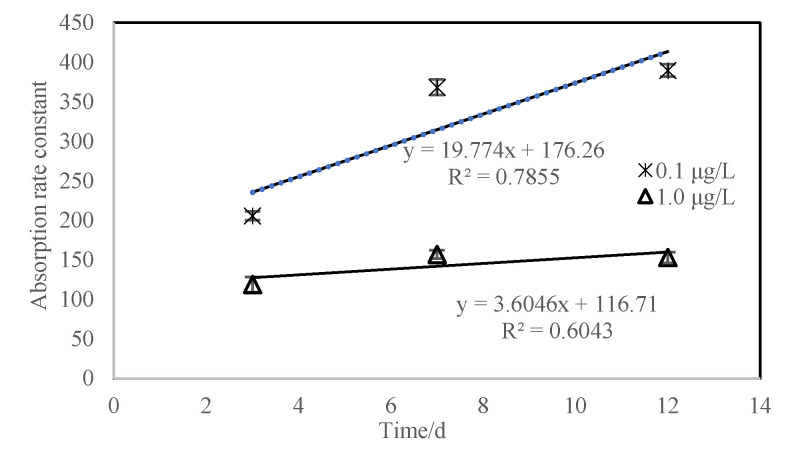
Concentration of pyraoxystrobin in zebrafish during exposure.

During the test, when the measured exposure concentration was 0.094 μg/L (the prepared concentration was 0.1 μg/L) and 0.94 μg/L (the prepared concentration was 1.0 μg/L), the average *BCF*_ss_ of the test substance was 820.8 and 265.9, respectively. When the exposure concentration was 0.094 μg/L, the absorption rate constant (*K*_1_) was 391.0 d^−1^; when the exposure concentration was 0.94 μg/L, the absorption rate constant (*K*_1_) was 153.2 d^−1^ (see Figure 3). 

### 3.3. The Results of the Clearance Test of Pyraoxystrobin in Fish

**Figure 4:**
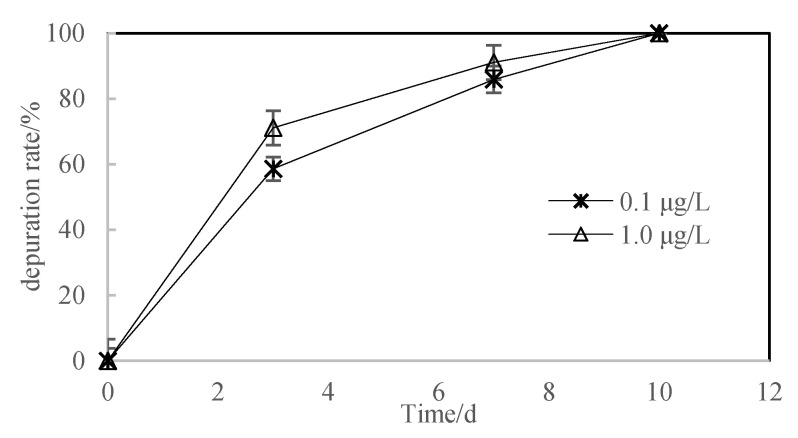
The clearance rate of pyraoxystrobin in zebrafish at the cleanup phase.

After 28 days of exposure to pyraoxystrobin, the zebrafish were transferred to clean water for the cleanup phase test. The results of the elimination phase test indicated that the test substance was quickly eliminated in the fish. On the third day, the concentration of pyraoxystrobin in the fish decreased by 58.6% and 71.1%, and by 85.9% and 91.1% on the 7th day. By the 10th day, the concentrations were all below the detection limit, with a clearance rate of 100% (see Figure 4).

**Figure 5:**
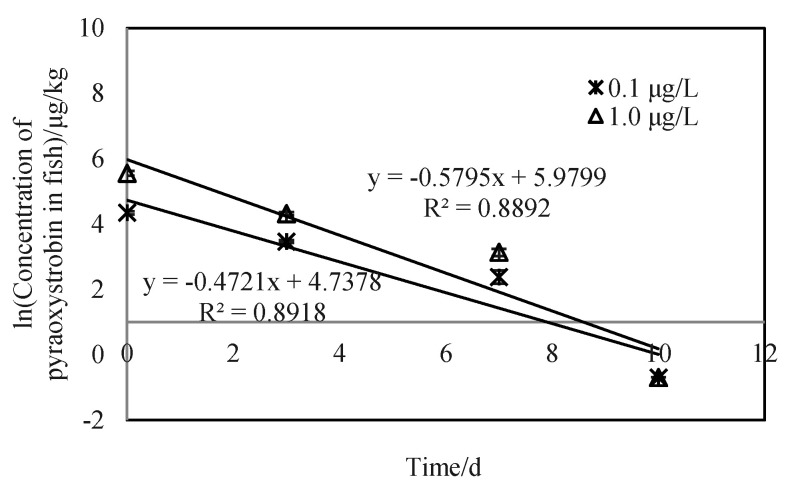
Clearance rate of pyraoxystrobin in zebrafish at the cleanup phase.

The data in Figure 5 showed that the elimination kinetics of pyraoxystrobin by zebrafish follows a first-order equation. Figure 4 showed the clearance rate of ln (pyraoxystrobin residue in zebrafish) over time. The clearance rate constants (*K*_2_) of pyraoxystrobin in zebrafish were 0.5795 and 0.4721, and the half-lives were 3.84 d and 3.33 d.

### 3.4. Dynamic Bioconcentration Factor

According to the test results, when the concentration was 0.1 μg/L, the dynamic enrichment factor was 828.4; when the concentration was 1.0 μg/L, the dynamic enrichment factor was 264.1.

### 3.5. BCF Model Estimation of Pyraoxystrobin

The concentration of pesticides in organisms is closely related to their water solubility, fat solubility, octanol/water partition coefficient, and soil adsorption partition coefficient. We calculated the *BCF* value of pesticides based on the physical and chemical properties of pesticides [10]. We estimated based on the compound *K*_ow_ value:

log*BCF* = 0.76log*K*_ow_ − 0.23 (2)

The log*K*_ow_ of pyraoxystrobin is 4.37 [5]. According to Formula (2), the estimated value of the bioconcentration coefficient *BCF* of pyraoxystrobin was 1233.

Estimation of *BCF* from water solubility: the estimation formula was deduced by Kenaga [11] in the laboratory by studying different fish species and 36 kinds of organic matter as follows:

log*BCF* = −0.564log*S* + 2.791 (3)

According to Formula (3) and the water solubility of pyraoxystrobin at 29 °C, the bioconcentration coefficient *BCF* value can be calculated to be 1953.1. 

We estimated *BCF* from the soil adsorption partition coefficient: There is an empirical relationship between *K*_oc_ and *BCF*. The affinity of soil for certain organic matter may be related to the affinity of the compound with certain parts of the ecosystem. Kenaga [11] derived the following estimation formula from the measured value of a small amount of soil adsorption partition coefficient:

log*BCF* = 1.12log*K*_oc_ − 1.58 (4)

The *K*_oc_ of pyraoxystrobin is 2905.9 [12]. According to Formula (4), the estimated value of the bioconcentration coefficient *BCF* of pyraoxystrobin is 398.

## 4. Discussion

Fish bioaccumulation refers to the ability of pollutants from the water environment to enter the fish body and accumulate. Usually, the bioconcentration factor (*BCF*) is used to characterize the tendency of contaminants to accumulate in fish. The index used to describe the enrichment effect of fish on pollutants is one of the important indicators for evaluating the environmental and health risks of pollutants. The stronger the bioaccumulation of pollutants is, the greater the degree of pollution and chronic harm to organisms will be [13,14]. It can be seen from Figure 1 that under the exposure conditions of pyraoxystrobin, the *BCF* of zebrafish basically reached a steady state on the 12th day. Calculated based on the concentration of the test substance in the test solution, the steady-state *BCF*_ss_ were 820.8 (714.8~925.3, 0.1 μg/L) and 265.9 (189.7~44.5, 1.0 μg/L), respectively. This study found that the accumulation of pyraoxystrobin in fish has no proportional relationship with the concentration in the corresponding water body, and does not increase proportionally due to the increase in exposure concentration. Franke [15] studied the enrichment characteristics of phenol in fish. He found that when the exposure concentration was 60 mg/L, the *BCF* value was 1.9; and when the exposure concentration dropped to 32.7 μg/L, the *BCF* value rose to 4312. Meanwhile, Xu [16] found that the sulfa antibiotics sulfamethazine and sulfamethoxazole were most enriched in zebrafish under low concentration exposure. Wu [17] found that the fluazinam were most enriched in zebrafish under low concentration exposure. The studies of Nallani [18] indicated that the enrichment of drugs in the environment by organisms is not boundless, but has a certain limit. When a certain threshold is reached, the digestion (or excretion) speed of the drug in the organism is equivalent to the absorption speed, that is, the enrichment balance is reached. The results of this experiment also proved that the concentration of pyraoxystrobin in the zebrafish under low-concentration exposure conditions is greater than that under high-concentration exposure conditions. According to the classification of bioaccumulation in Test Guidelines on Environmental Safety Assessment for Chemical Pesticides, pyraoxystrobin is moderately enriched.

From the results calculated by the three prediction models in this experiment, it can be seen that the *BCF* values estimated by the three prediction models predicted *BCF* values that were generally larger than the measured results, but in the same range of magnitude. This indicates that the bioaccumulation of pyraoxystrobin in fish is related to *K*_ow_, water solubility, and *K*_oc_ value. The enrichment of pyraoxystrobin in organisms follows the characteristics of the hydrophobic model, that is, the logarithm of the migration process of the drug into the organism increases with the increase in drug hydrophobicity [19]. Figure 3 and Figure 4 showed the elimination kinetic rate of pyraoxystrobin in zebrafish, which is consistent with the existing reports on complex metabolism in fish [20,21]. The clearance rate constants (*K*_2_) of pyraoxystrobin in zebrafish were 0.5795 and 0.4721, and the half-lives were 3.84 and 3.33 d, respectively. At present, studies have reported that quinalphos-carp (half-life of 1.9 d) and silver barb (half-life of 2.5 d) [22], carbofuran has a half-life of 1.63 d in zebrafish), and carbofuran in zebrafish has a half-life of 3.33 d [23], the half-life of fipronil in rainbow trout is 0.61 d [24], and the half-life of fipronil in loach is 1.8–3.8 d [25], which is close to the results of this study.

## 5. Conclusions

The bioaccumulation effect of pyraoxystrobin on zebrafish was found to be relatively slow, and it took 12 days to reach a stable state of equilibrium. Based on the General Administration of Quality Supervision, Inspection and Quarantine of the People’s Republic of China [26], pyraoxystrobin was moderately enriched. Pyraoxystrobin has high toxicity and certain bioaccumulation to aquatic organisms. The comprehensive evaluation believes that pyraoxystrobin is highly enriched in surface water organisms and has potential pollution risks to water organisms.

## Figures and Tables

**Table 1:** Gradient elution program.

Time/min	Mobile Phase A/%	Mobile Phase B/%
0.0	40	60
0.6	95	5
2.3	95	5
2.31	40	60
3.30	40	60

**Table 2:** Concentration of tested solution and fish body.

Time/d	Treat	Water μg/L	Fish μg/kg	Treat	Water μg/L	Fish μg/kg
0	0.1 μg/L	0.091	–	1.0 μg/L	0.92	–
3		0.095	41.3		0.93	191.6
7		0.094	73.1		0.93	252.3
12		0.096	79.5		0.99	260.2
18		0.091	74.8		0.93	249.4
22		0.091	74.3		0.93	245.6
28		0.095	77.6		0.93	260.8

## Data Availability

Not applicable.

## References

[1] Li M., Liu C., Li Z.L., Zhang H., Si N. (2011). The discovery of fungicide pyraoxystrobin. Agrochemicals.

[2] Li M., Yang J., Liu C. (2014). Fungicide-pyraoxystrobin. World Pestic..

[3] Chen L., Liu J., Si N. (2011). Pyraoxystrobin and epoxiconazole against rice blast. Agrochemicals.

[4] Wang L., Li B., Xiang W., Shi Y., Liu C. (2008). Control effects of Pyraoxystrobin on cucumber powdery mildew. Agrochemicals.

[5] Yang T., Xu C., Liu X., Chen X., Zhang J., Ding X. (2014). Fate of a Novel Strobilurin Fungicide Pyraoxystrobin in Flooded Soil. Environ. Sci. Process. Impacts.

[6] Liu X., Wu H., Hu T., Ding X. (2018). Adsorption and leaching of novel fungicide Pyraoxystrobin on sSoils by 14C tracing method. Environ. Monit. Assess..

[7] Chen L., Song Y., Tang B., Song X., Yang H., Li B., Zhao Y., Huang C., Han X., Wang S. (2015). Aquatic Risk Assessment of a novel strobilurin fungicide: A microcosm study compared with the species sensitivity distribution approach. Ecotoxicol. Environ. Saf..

[8] Li H., Yu S., Cao F., Wang C., Zheng M., Li X., Qiu L. (2018). Developmental Toxicity and Potential Mechanisms of Pyraoxystrobin to Zebrafish (*Danio rerio*). Ecotox. Environ. Saf..

[9] Yang X.H., Liu H.J., Jia M.H., Wang J.F., Wu J.L., Song J.H., Liu Y.X. (2020). Evaluation of pyraoxystrobin bioconcentration in zebrafish (*Danio*
*rerio*) using modified QuEChERS extraction. J. Environ. Sci. Health Part B.

[10] Arnot J., Gobas F. (2006). A review of bio-concentration factor (BCF) and bioaccumulation factor (BAF) assessments for organic chemicals in aquatic organisms. Environ. Rev..

[11] Kenaga E., Goring C. (1980). Relationship between water solubility, soil sorption, octanol/water partitioning, and concentration of chemicals in biota. Aquatic Toxicology.

[12] Liu X. (2014). Synthesis of a 14C Labeled Novel Fungicide Pyraoxystrobin and Its Environmental Behavior and Fate in Soil.

[13] Hilvarsson A., Ohlauson C., Blanck H., Granmo A. (2009). Bioaccumulation of the new antifoulant medetomidine in marine organisms. Mar. Environ. Res..

[14] Li L., Jiang M., Shen X., Wang Y., Xu G. (2015). Kinetic study of the bioaccumulationof benzo[a]pyrene in two sea creature. China Environ. Sci..

[15] Franke C. (1996). How meaningful is the bio-concentration factor for risk assessment?. Chemosphere.

[16] Xu J., Wang N., Kong D., Kong X., Shan Z. (2015). Bioconcentration of Sulfonamide Antibiotics in Zebrafish (*Brachydanio rerio*) and Model Prediction Assessment. Asian J. Ecotoxicol..

[17] Wu W., Kong D., Zhang W., Bu Y., Li J., Shan Z. (2020). Acute toxicity of fluazinam to aquatic organisms and its bioaccumulation in *Brachydanio rerio*. Environ. Sci. Pollut. Res..

[18] Nallani G., Paulos P., Constantine L., Venables B., Huggett D. (2011). Bioconcentration of ibuprofen in fathead minnow (*Pimephales promelas*) and channel catfish (*Ictalurus punctatus*). Chemosphere.

[19] Veith G., Defoe D., Bergstedt B. (1979). Measuring and estimating the bioconcentration factor of chemicals on fish. J. Fish Res. Board Can..

[20] Ulhaq M., Sundstrom M., Larsson P., Gabrielsson J., Bergman A. (2015). Tissue uptake, distribution and limination of (14)C-PFOA in zebrafish (*Danio rerio*). Aquat. Toxicol..

[21] Steinbach C., Grabic R., Fedorova G., Koba O., Kroupova H. (2016). Bioconcentration, metabolismand half-Life time of the human therapeutic drug diltiazem in rainbow trout oncorhynchus mykiss. Chemosphere.

[22] Thinh N., Phu T., Douny C., Phuong N., Huong D., Kestemont P., Scippo M. (2018). Bioconcentration and half-life of quinalphos pesticide in rice-fish integration system in the Mekong Delta, Vietnam. J. Environ. Sci. Health Part B.

[23] Cui J., Wang F., Gao J., Zhai W., Zhou Z. (2019). Bioaccumulation and metabolism of carbosulfan in zebrafish (*Danio rerio*) and the toxic effects of its metabolites. J. Agric. Food Chem..

[24] Konwick B., Garrison A., Black M., Avants J., Fisk A. (2006). Bioaccumulation, biotransformation, and metabolite formation of fipronil and chiral legacy pesticides in rainbow trout. Environ. Sci. Technol..

[25] Gao J., Wang F., Jiang W., Han J., Liu D., Zhou Z., Wang P. (2019). Tissue distribution, accumulation, and metabolism of chiral flufiprole in Loach (Misgurnus anguillicaudatus). J. Agric. Food Chem..

[26] (2014). General administration of quality supervision, inspection and quarantine of the People’s Republic of China (GAQSIQ). Test Guidelines on Environmental Safety Assessment for Chemical Pesticides-PART 7: Bioconcentration Test.

